# Correlation between learning styles and attitudes toward interprofessional education among medical students: a cross-sectional study

**DOI:** 10.1186/s12909-024-05710-w

**Published:** 2024-09-16

**Authors:** Chengrui Zhang, Xinxin Wang, Ying Xing, Wei Luan, Tao Jin

**Affiliations:** 1 Shanghai Jiao Tong University School of Nursing, Shanghai, China; 2 Shuguang Hospital, Shanghai University of TCM, Shanghai, China

**Keywords:** Attitude, Collaborative learning, Interprofessional learning, Learning styles, Medical Education Research, RIPLS, Students

## Abstract

**Background:**

Interprofessional teamwork improves patient care quality, safety, and health outcomes. Interprofessional education (IPE) is crucial in today’s medical education to prepare students for the workforce as integral members of a collaborative team. The diversity of IPE learners indicates the importance of exploring the relationship between learning styles and attitudes toward IPE. The purpose of this study was to investigate the relationship between learning styles and attitudes toward IPE.

**Methods:**

A cross-sectional study was conducted between August 2023 and September 2023 in 49 colleges located in the south-eastern region of China. A convenience sampling approach was employed, selecting 500 students majoring in Clinical Medicine and Nursing. The students completed an online questionnaire, which included sociodemographic characteristics, educational characteristics, interprofessional educational characteristics, learning styles, and the readiness for interprofessional learning scale, and Kolb’s learning style inventory. Descriptive statistics, Spearman’s correlation, and multiple linear regression analysis were used to analyze the data.

**Results:**

The most learners are diverger (93.2%), followed by assimilator (3.4%), accommodator (2.6%), and Converger (0.8%). The total score on the RIPLS was 69.70 (7.42), ranging from 48 to 88. A statistical relationship could be established between learning styles and attitudes toward IPE.

**Conclusion:**

Abstract conceptualization and active experimentation learning modes and convergers were closely linked with positive attitudes toward IPE. Gender, age, and study stress can affect attitudes toward IPE. This study highlights the need for medical education curricula to integrate innovative teaching methods such as PBL, role-playing, scenario simulation and clinical early exposure to strengthen professional identity, and improve abilities related to interprofessional learning.

## Introduction

Interprofessional education (IPE) has emerged as a critical educational approach for addressing the growing complexity of healthcare and various professional fields. The World Health Organization (WHO) defines IPE as an educational approach in which students from two or more professions learn about, from, and with each other [1]. IPE can prepare students for the workforce as individuals in their respective fields and as integral members of a collaborative team [2]. IPE is crucial in today’s medical education where medical students are educated in silos and of teamwork and collaboration [3]. With these capacity developments, IPE is seen as a key step in advancing healthcare, where the collective expertise of different professionals is leveraged to provide the best possible patient care quality, safety, and health outcomes​ [4, 5].

Learning styles are characteristic cognitive, effective, and psychosocial behaviors that serve as relatively stable indicators of how learners perceive, interact with, and respond to the learning environment [6]. Kolb’s experiential learning theory describes learning as a continuous cycle of four learning modes [7]. This cycle starts with the concrete experience (CE) mode, followed by the reflective observation (RO) mode, the abstract conceptualization (AC) mode, and the active experimentation (AE) mode [8]. Learners use the CE mode for experience, the RO mode for reflection, the AC mode for thinking, and the AE mode for action [9]. Kolb found that learners typically did not use all 4 learning modes equally but preferred to concentrate on one or two of them [10]. Therefore, the learning cycle can be divided into 2 dimensions. The primary dimension is AC-CE, which measures the balance between the AC and the CE mode. The AC-CE dimension can influence learners’ preference to learn and interact with new knowledge. When AC-CE is positive, it suggests that the learner favors the AC mode more than the CE mode. Likewise, the other dimension is AE-RO, which plays a crucial role in how learners transform and apply knowledge [11]. Based on Kolb’s Experiential Learning Theory, Kolb’s Learning Style Inventory (LSI) classifies learning styles into 4 types: accommodators, divergers assimilators and convergers [12]. Accommodators are learners who actively engage in new experiences and adapt well to changes, whereas divergers view concrete situations from multiple perspectives and are creative. Assimilators are inductive thinkers who create theoretical models, and convergers apply practical ideas and perform well in problem-solving scenarios. These styles guide the learning process in different ways [9, 13].

IPE is characterized by the diversity of its learners, stemming from various backgrounds and disciplines, thus resulting in different learning styles [14]. This diversity underscores the necessity for more flexible teaching approaches tailored to accommodate different learners, to maximize the educational effectiveness [15]. Beforehand, this diversity indicates the importance of exploring the relationship between learning styles and attitudes toward IPE, because attitude toward IPE forms the foundation for engaging in IPE [16]. However, current research on the correlation between learning styles and attitudes toward IPE are limited by small, region-specific sample sizes, underscoring the need for more extensive, globally diverse research to better comprehend these relationships [17, 18]. The primary aim of this study was to investigate the relationship between learning styles and attitudes toward IPE. This study endeavors to provide a robust theoretical foundation for the development of IPE strategies based on learning style to help educators enhance the overall effectiveness of IPE among medical students.

## Methods

The reporting of the study followed Strengthening the Reporting of Observational Studies in Epidemiology (STROBE) Statement guidelines [19].

### Study design and participants

A cross-sectional study was comducted from August 2023 to September 2023, using a convenient sampling method. Students majoring in Clinical Medicine (5-year program) and Nursing (4-year program) were recruited from 49 colleges in the southeast region of China, comprising 37 comprehensive universities and 12 independent medical schools, across 30 cities. The inclusion criteria were as follows: students enrolled in full-time education; students voluntarily participated in the survey. The exclusion criteria were as follows: students in a junior college to undergraduate upgrading program.

The sample size was calculated based on the following formula: $N = \frac{{({{(f \times z(\frac{a}{2}) + z(\beta ))}^2} \times (k - 1)}}{{[{f^2} \times (k - 1)]}}$, where α is the significance level, which was set to 0.05, and the statistical power was set to 0.80. $Z = (\frac{\alpha }{2})$ is the coefficient corresponding to the confidence level under a normal distribution, $Z = (\frac{0.05}{2})$ is 1.96, and $Z = (\frac{0.08}{2})$ is 0.84. K was set to 1, and f was set to 0.20, resulting in *N* = 436. Considering a 20% dropout rate, the required number of students was *N* = 523.

### Data collection

The study adopted online questionnaire survey. The researchers contacted teachers from the 49 medical colleges with which the research team had established collaboration, to distribute the Wenjuanxing online questionnaire and provide online instructions. Students were invited to complete the anonymous survey within 2 weeks.

### Measures

#### Attitudes toward IPE

The study employed the readiness for interprofessional learning scale, which was originally developed by PARSELL et al. and translated into Chinese by Wang Xiyi et al. [20]. This scale is utilized to assess medical students’ attitudes toward IPE. The instrument consists of 4 dimensions—team cooperation, negative professional identity, positive professional identity, and roles and responsibilities—for a total of 19 items. The students rated their agreement with each statement on a 5-point Likert scale ranging from “strongly disagree” (1) to “strongly agree” (5). For items related to negative professional identity and roles and responsibilities, reverse scoring was applied. The composite score spans from 19 to 95, with higher scores denoting more active attitude towards IPE.

#### Kolb’s learning style inventory

The study employed the Kolb’s Learning Style Inventory (LSI) version 3.1 questionnaire, which was translated into Chinese by Tan et al. [21]. Kolb’s LSI comprises 12 items, and participants are required to rate each item on a scale ranging from 1 to 4, reflecting their learning preferences. This assessment tool is grounded in Kolb’s learning theory. Scores for each of these learning modes are cumulative, yielding a range of 12 to 48 points, with higher scores signifying a greater preference for the specific learning mode. Kolb’s LSI classifies learners into 4 distinctive learning styles: the accommodator, the diverger, the assimilator, and the converger.

#### Related variables

Sociodemographic characteristics obtained: age, gender, grade, only child. Grade included Grade 1 ~ Grade 5. ‘Only child’ was identified using the question: “Are you the only child in your family?” and was divided into “only child” and “not the only child”.

Educational characteristics obtained: school type, discipline, academic performance, study stress, discipline as the first choice. School type was classified into “comprehensive university” and “Independent medical school”. Discipline was categorized as “clinical medicine” and “nursing”. Academic performance was evaluated using the question, “What’s your ranking in the school?”. The response options were as follows: “top 30%”, “top 50%”, “bottom 50%”, “bottom 30%”. Study stress was evaluated using the question, “What is your self-assessed study stress?”, and categorized into “very low”, “low”, “average”, “high”, and “very high”. “Discipline as the first choice” was determined using the question, “Is your current discipline (clinical medicine/nursing) the one you chose as your first choice in the college entrance examination?”. The response options were “yes” or “no”.

Interprofessional educational characteristics obtained: previous interprofessional study experience, willingness to participate in interprofessional studies, willingness to participate in interprofessional discussions. Previous interprofessional study experience was asked using the question, “Have you ever had any interprofessional study experience?”, and the answer was “yes” or “no”. Willingness to participate in interprofessional studies was evaluated using the question “Would you like to participate in interprofessional studies?”, and the answer was categorized into “very unwilling” (1) to “very willing” (5). “Willingness to participate in interprofessional discussions” was evaluated using the question “Are you willing to share your knowledge or skills with people from different disciplines?”, and the answer was categorized into “very unwilling” (1) to “very willing” (5).

### Statistical analysis

An excel sheet was automatically generated from the Wenjuanxing online system, and statistical analyses were performed using the SPSS software (version 24.0). The normal distribution of the data was evaluated using the Kolmogorov–Smirnov test. Descriptive statistics were used to describe the data. The sociodemographic characteristics, educational characteristics, and interprofessional educational characteristics differences in attitudes toward interprofessional learning were assessed using t-test and ANOVA test. The correlations between learning styles and attitudes toward IPE were assessed using Pearson correlation analysis. The influencing factors were determined using multiple stepwise linear regression analysis in which the residual showed a normal distribution. A p value of < 0.05 was considered significant.

### Ethical considerations

The study received approval from the Shanghai Jiao Tong University School of Medicine, Renji Hospital Ethics Committee (reference number: RA-2021-465) and adhered to the principles of the Declaration of Helsinki. The questionnaire’s cover page outlined the study’s purpose, voluntary participation, confidentiality measures, and implied consent upon submission.

## Results

### Baseline characteristics, learning styles, and attitudes toward IPE among medical students

Of the 523 questionnaires collected in total, 500 were suitable for analysis (a response rate of 95.60%). The mean age of the students was 20.78 (1.29), ranging from 18 to 24. 54.80% were male, 50.00% were clinical medicine students, 50% were nursing students, 66.80% had previous interprofessional study experience. 90.40% had top 50% academic performance. 84.60% of the students were willing to participate in interprofessional studies, and 85.00% were willing to participate in interprofessional discussions.

The distribution of learning styles among the students showed that most learners are divergers (93.2%), followed by assimilators (3.4%) and accommodators (2.6%). Convergers(0.8%) are the rarest learners.

The total score on the RIPLS was 69.70 (7.42), ranging from 48 to 88. For the 4 subscales, the teamwork and collaboration (items 1–9) score was 38.29 (4.64), ranging from 18 to 45; the negative professional identity (items 10–12) score was 8.47 (3.99), ranging from 3 to 15. The average positive professional identity score (items 13–16) was 16.73 (2.86), ranging from 5 to 20; the average role and responsibility score (items 17–19) was 6.21 (1.99), ranging from 3 to 13.

### Analysis of the influencing factors of attitudes toward IPE

The results of the t-test and ANOVA revealed statistically significant (*p* < 0.05) differences in the students’ attitudes toward IPE in terms of gender, school type, academic performance, self-assessment of study stress, willingness to participate in interprofessional studies, and willingness to participate in interprofessional discussions (see Table 1).

**Table 1:** Influencing factors of attitudes toward IPE (*n* = 500)

Characteristic	*N*	Mean	SD	t/F	*P* Value
**Gender**				-3.951	**< 0.01**
Male	274	68.52	7.470		
Female	226	71.12	7.124		
**School type**				2.018	**0.044**
Comprehensive university	391	70.05	7.218		
Independent medical school	109	68.43	8.019		
**Discipline**				0.687	0.493
Clinical medicine	250	69.92	7.317		
Nursing	250	69.47	7.534		
**Only child**				0.857	0.392
Yes	320	69.91	7.256		
No	180	69.32	7.715		
**Discipline is the first choice**				1.602	0.115
Yes	448	69.92	7.141		
No	52	67.77	9.367		
**Have previous interdisciplinary study experience**				-1.827	0.068
Yes	334	69.27	7.158		
No	166	70.55	7.878		
**Grade**				1.987	0.095
Grade 1	22	69.14	6.847		
Grade 2	135	68.51	7.380		
Grade 3	206	70.25	7.496		
Grade 4	108	70.67	7.399		
Grade 5	29	68.10	7.053		
**Academic performance**				4.381	**0.050**
Top 30%	207	71.04	7.054		
Top 50%	249	68.85	7.617		
Bottom 50%	42	68.43	7.195		
Bottom 30%	2	63.00	4.243		
**Study stress**				3.151	**0.014**
Very low	12	74.92	6.403		
Low	22	72.50	8.456		
Average	122	68.50	7.885		
High	258	69.68	7.205		
Very high	86	70.00	6.850		
**Willingness to participate in interprofessional studies**				19.435	**< 0.01**
Very Unwilling	2	59.50	16.263		
Unwilling	9	61.67	9.747		
Generally	66	65.48	7.873		
Willing	212	68.73	6.944		
Very willing	211	72.43	6.373		
**Willingness to participate in interprofessional discussions**				29.026	**< 0.01**
Very Unwilling	4	63.75	10.720		
Unwilling	11	58.36	5.870		
Generally	60	64.47	6.624		
Willing	194	68.72	6.948		
Very willing	231	72.52	6.484		

### Correlation analysis of attitudes toward IPE

Correlation analysis showed that there was a significant correlation between learning styles and attitudes toward IPE, and no multicollinearity was detected. RIPLS scores were associated with CE (*r* = 0.362, *p* < 0.01), RO (*r* = 0.474, *p* < 0.01), AC (*r* = 0.465, *p* < 0.01), AE (*r* = 0.506, *p* < 0.01), and AC-CE (*r* = 0.146, *p* < 0.01) (see Table 2).

**Table 2:** Correlation analysis of attitudes toward IPE (*n* = 500)

	M	SD	CE	RO	AC	AE	AE-RO	AC-CE	RIPLS
CE	39.67	4.95	1						
RO	40.51	4.95	0.792**	1					
AC	40.20	4.93	0.764**	0.816**	1				
AE	40.58	4.86	0.759**	0.825**	0.823**	1			
AE-RO	0.07	2.91	-0.081	− 0.325**	-0.014	0.266**	1		
AC-CE	0.52	3.39	− 0.350**	0.029	0.336**	0.088*	0.099*	1	
RIPLS	69.70	7.42	0.362**	0.474**	0.465**	0.506**	0.038	0.146**	1

Note. M, mean; SD, standard deviation; CE, concrete experience; RO, reflective observation; AC, abstract conceptualization; AE, active experimentation

### Multifactor analysis of attitudes toward IPE

Multiple stepwise linear regression analysis was conducted with the RIPLS score as the dependent variable, all sociodemographic characteristics, educational characteristics, interprofessional educational characteristics and learning style variables as the independent variable. The results revealed statistically significant differences in RIPLS scores between different genders, age, learning styles, AE score, AE-RO score, willingness to participate in interprofessional discussions, study stress (*p* < 0.05) (see Table 3).

**Table 3:** Multifactor analysis of attitudes toward IPE (*n* = 500)

Characteristic	B	SE(B)	β	t	*P*	95%CI	
**Constant**	21.764	7.659	-	2.841	**0.005**	6.714	36.814
**Gender**							
Female	-	-	-	-	-	-	-
Male	-1.348	0.580	-0.090	-2.325	**0.020**	-2.487	-0.209
**School type**							
Comprehensive university	-	-	-	-	-	-	-
Independent medical schools	-0.484	0.667	-0.027	-0.725	0.469	-1.795	0.828
**Discipline**							
Medicine	-	-	-	-	-	-	-
Nursing	-0.401	0.588	-0.027	-0.683	0.495	-1.556	0.753
**Grade**							
Grade 2	-	-	-	-	-	-	-
Grade 1	0.686	1.416	0.019	0.484	0.628	-2.096	3.468
Grade 3	0.635	0.848	0.042	0.749	0.454	-1.031	2.300
Grade 4	-0.341	1.187	-0.019	-0.287	0.774	-2.675	1.992
**Only child**							
Yes	-	-	-	-	-	-	-
No	0.366	0.578	0.024	0.633	0.527	-0.770	1.503
**Discipline as the first choice**							
Yes	-	-	-	-	-	-	-
No	0.546	0.906	0.022	0.603	0.547	-1.234	2.326
**Have previous interprofessional study experience**							
Yes	-	-	-	-	**-**	-	-
No	1.554	0.581	0.099	2.674	**0.008**	0.412	2.695
**Learning style**							
Diverger	-	-	-	-	-	-	-
Accommodator	3.258	1.952	0.070	1.669	0.096	-0.578	7.094
Converger	11.226	3.266	0.135	3.438	**0.001**	4.810	17.643
Assimilater	2.027	1.696	0.050	1.196	0.232	-1.305	5.359
**Age**	0.743	0.372	0.129	1.994	**0.047**	0.011	1.474
**AE**	0.643	0.068	0.421	9.476	**0.000**	0.510	0.776
**AE-RO**	-0.370	0.115	-0.145	-3.219	**0.001**	-0.596	-0.144
**AC-CE**	0.103	0.093	0.047	1.098	0.273	-0.081	0.286
**Willingness to participate in interprofessional discussions**	2.107	0.373	0.232	5.643	**0.000**	1.373	2.840
**Study stress**	-0.700	0.313	-0.082	-2.235	**0.026**	-1.315	-0.085

Note: R^2^ = 0.359, _adj_R^2^= 0.359, F = 15.68, *p* < 0.01, Durbin–Watson = 1.934, no collinearity

B: No standardized coefficient; SE: Standard error; β: Standardization coefficient; R: Coefficient of determination; CE, concrete experience; RO, reflective observation; AC, abstract conceptualization; AE, active experimentation

## Discussion

This study offered insights into whether learning styles might affect attitudes toward IPE . A statistical relationship could be established between learning styles and attitudes toward interprofessional collaboration, which is not in line with previously reported research [6].

### Students’ attitudes toward IPE

In this study, the total RIPLS score was 69.70, which is lower than that reported in previous studies carried out in China, which ranged from 70.49 ~ 73.55 [9, 10]. This difference can be explained by the difference in sample selection. It was reported that attitudes toward IPE change as student grades and discipline change [11]. The study excluded disciplines such as public health and physical therapy, which were associated with higher RIPLS scores. This study included only nursing students and clinical medicine students because collaboration between nurses and clinicians is the most common collaboration in clinical environments and directly affects patient outcomes [12].

The scores for the subscales negative professional identity and roles and responsibility were 8.47 and 6.21, respectively, which were both lower than those of previous studies, with scores ranging from 10.35 ~ 11.15 and 7.72 ~ 9.74, respectively. The scores for the teamwork and collaboration subscale and for positive professional identity were 38.29 and 16.73, respectively, which were both higher than those of previous studies, with scores ranging from 36.13 ~ 37.21 and 15.97 ~ 16.07, respectively [9, 10]. The results revealed students’ poor understanding of their professional role. This could be because the students selected were mainly in grades 2 ~ 4 and were not directly aware of real-world doctor and nurse cooperation [13]. In the future, medical education should pay more attention to early exposure to clinical settings and career guidance to remind students of the more precise positioning of doctors and nurses on the current interprofessional team [14].

### Relationships between gender, age, study stress and discipline and attitudes toward IPE

The present study showed that gender, age, and discipline can influence attitudes toward IPE. Female students had a more positive attitude toward IPE, which is consistent with the findings of previous research [17, 18]. This phenomenon could be explained that females usually have more trust in their team colleagues’ abilities but less confidence in their own abilities, as a result show more interest in teamwork, which is indispensable in IPE [22]. Older students had a more positive attitude toward IPE, which was also consistent with the findings of previous research [23]. Older students often have a deeper understanding of interprofessional cooperation and therefore obtain higher scores. Students with more academic stress had more negative attitudes toward IPE. Previous studies have reported that study stress results in sleep disorders, decreased attention, and increased interpersonal conflicts [24, 25]. These factors may lead to reduced attitudes towards IPE.

In contrast to the findings of previous research, the study found no significant difference in the attitudes of clinical medicine and nursing students toward IPE [26, 27]. For the past few years. the nurse‒doctor relationship has gradually shifted from the traditional dominant-subordinate model to a juxtaposition-complementary model, and students realize that skill enhancement is necessary, interprofessional cooperation is mutual [28].

### Relationships between learning modes and learning styles and attitudes toward IPE

#### Learning modes and attitudes toward IPE

Compared with the other 3 learning modes, the AE mode can increase enthusiasm toward IPE. The results was in line with previous research and together showed that AE-dominant learners (accommodators and convergers) were associated with more active attitudes toward IPE [17]. The study showed that learners who emphasized the AC mode over the CE mode (AC-CE) showed more active attitudes toward IPE. In conclusion, learners who prefer the AC and AE mode typically demonstrated more active attitudes toward IPE.

The AC mode requires learners to use logic to assimilate and distill their experience and reflections into a generalized concept [18]. Interprofessional learners use the AC mode mainly in the preparation phase before IPE, where the learners independently study interprofessional knowledge unfamiliar to them. The preparation phase often involves theoretical concepts and favors a more conceptual and analytical style of learning, aligning well with AE mode [29, 30]. This can explain why learners who emphasize AC over CE are more active. To improve learning outcomes for individuals who favor the CE mode, it is advisable to incorporate vivid clinical cases [31]. This strategy helps make the knowledge more tangible and relatable, thereby facilitating a deeper understanding and engagement with the interprofessional knowledge.

Learners who prefer the AE mode are more active in IPE because the mode can help make decisions and solve problems, corresponding to the competency framework required for IPE, which emphasis conflict resolution [9, 32]. In IPE study, learners solve problems related to real-world healthcare scenarios together with their interprofessional team members, apply prior knowledge to suggest and critique solutions [29, 30]. The AE mode is particularly vital in real-world healthcare scenarios where clinicians and nurses frequently encounter challenges such as communication conflicts and complex clinical issues [33]. To enhance AE mode, problem-based learning (PBL) approach can be adopted. This education method can not only sharpen critical thinking and decision-making skills but also help healthcare professionals effectively navigate and resolve real-life medical situations [34, 35].

### Learning style and attitudes toward IPE

The study showed that convergers had more active attitudes toward IPE. The converging learning style involves using the AC mode and AE mode. These results are in line with the relationship between learning mode and attitudes toward IPE in this study, and consistent with previous studies [17]. Convergers’ unique characteristics can explain their passion for IPE. Firstly, Convergers are known for their strong problem-solving skills, as they can approach situations logically and analytically [9]. This trait is particularly beneficial in interprofessional settings where complex, multifaceted problems often arise. Secondly, convergers are more adaptable to education innovations. For instance, a study integrating Facebook into traditional classroom settings showed that participants with a converging learning style performed better, highlighting their adaptability and openness to new teaching methods [36]. This adaptability is crucial in IPE, where learners must adapt and apply concepts from various disciplines.

The study highlights a significant gap in current interprofessional education. The study identifies convergers—learners who excel in both abstract conceptualization and active experimentation—as particularly adept at interdisciplinary learning yet markedly rare. The study serves as a pivotal call to action, not only aims to foster convergers but also enhances overall learners’ engagement and proficiency in handling diverse and multifaceted clinical problems, thereby preparing a more adaptable and innovative future workforce.

This study recommends 2 potentially effective strategies. Firstly, integrating role-playing and scenario simulation into regular curriculums can enhance IPE by clarifying roles and improving skills such as communication, teamwork, empathy, and mutual respect [37]. These strategies offer a structured and safe learning environment that boosts student competencies in IPE [38]. Secondly, PBL should be implemented as it develops problem-solving skills, and enhances learners’ engagement and motivation by fostering self-directed learning [39, 40]. These improvements in skills prepare learners effectively for IPE.

## Conclusion

The cross-sectional study investigated the relationship between Kolb’s learning styles and attitudes toward IPE. The study found that attitudes toward IPE are less positive than those reported in previous studies carried out in China, with a notable lack of clarity in team roles. Additionally, factors such as gender, age, and study stress can affect attitudes toward IPE.

The study identified that AC and AE modes, characteristic of convergers, are better for IPE, yet such learners are rare. These findings underscore the need for medical education curricula to adopt innovative teaching methods like clinical early exposure, PBL, role-playing, and scenario simulation. These methods can strengthen students’ professional identity and clarify their roles, while enhance collaborative skills and readiness for interprofessional healthcare environments.

### Strengths and limitations

The study benefits from a substantial sample size, which enhances the statistical power and generalizability of the findings. By including a diverse range of student from 49 colleges across 30 cities within the south-eastern region of China, the study captures a broader representation of the population. This study is inconsistent with results of previous studies but provide evidence to demonstrate the potential association between learning styles and attitudes towards IPE, Paving the way for future studies. One limitation of this study is the cross-sectional design, which restricts its ability to establish causal relationships. Since data is collected at a single point in time, it cannot determine whether learning styles influence attitudes towards IPE or vice versa. Longitudinal studies would be necessary to explore the temporal aspects of this relationship. Another limitation is the use of convenience sampling, which can introduce selection bias. Future studies should consider using random sampling to enhance the representativeness and generalizability of the study results.

## Data Availability

The data related to this study are available from the corresponding author on reasonable request and pending additional ethical approval.
